# Supplementary material for: Polyacrylonitrile/Aminated Polymeric Nanosphere Nanofibers as Efficient Adsorbents for Cr(VI) Removal
Source: Molecules. 2022 Oct 21;27(20):7133. doi: 10.3390/molecules27207133 (PMC9611933; doi:10.3390/molecules27207133)
Supplement: Supplementary file 1 [file molecules-27-07133-s001.zip › molecules-1972571-supplementary.pdf]

Supplementary data

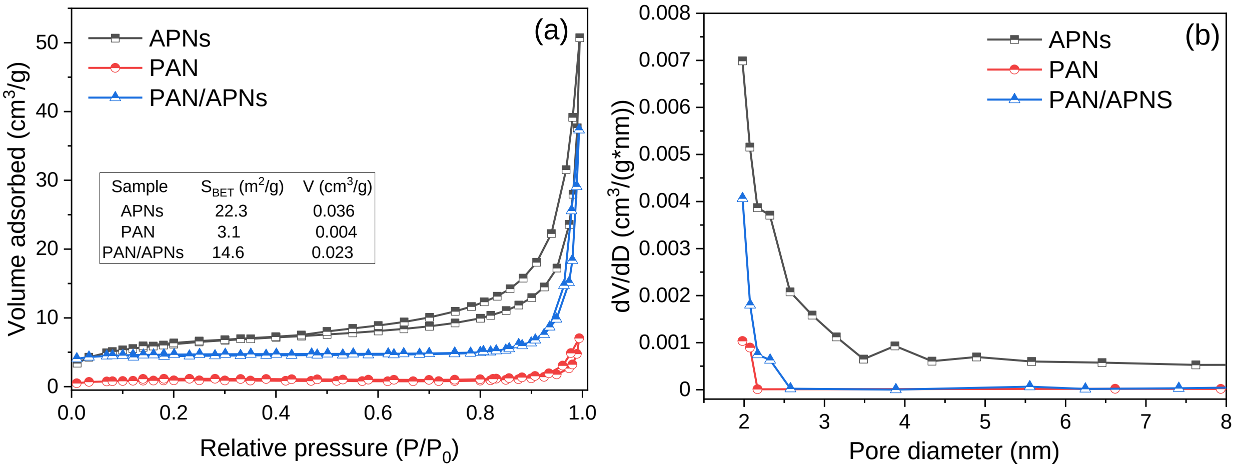

Figure S1. Nitrogen adsorption-desorption isotherms (a) and pore size distribution curves (b) of APNs, PAN and PAN/APNs.

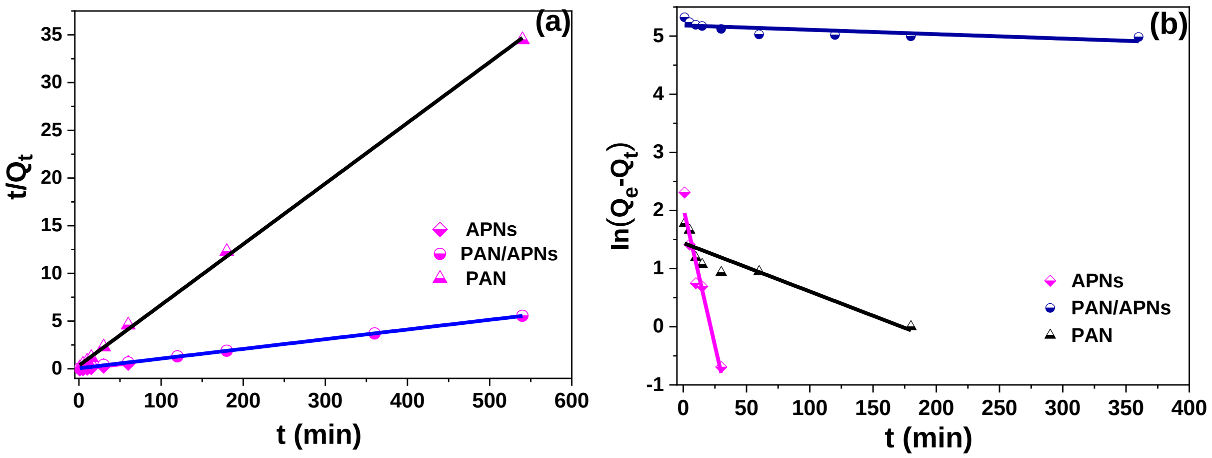

Figure S2. The fitting kinetic data of pseudo second order (a), pseudo first order (b).

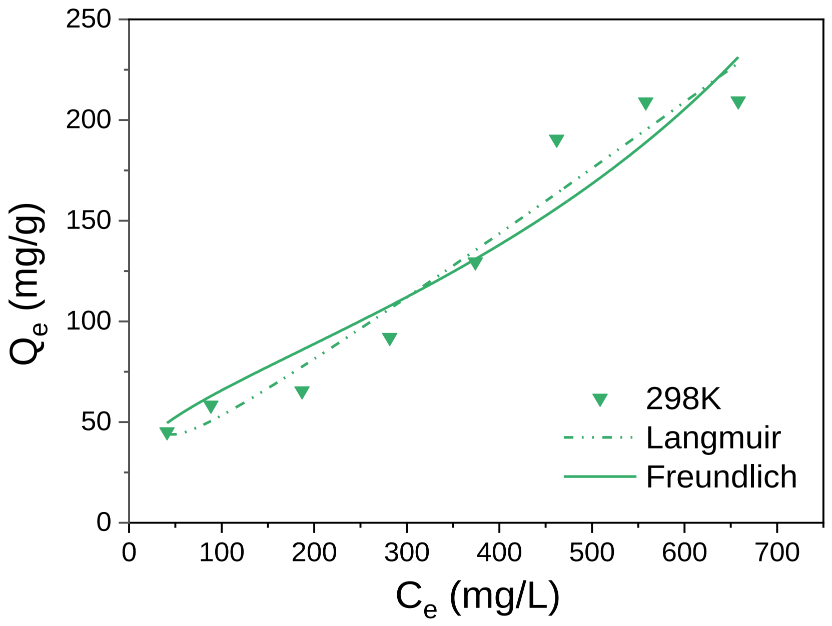

Figure S3. Langmuir and Freundlich isotherm models for Cr(VI) adsorption onto PAN. (pH = 2, adsorbent dose = 0.2 g/L).

Table S1. The pseudo second order and the first order model parameters

Samples	Q_{exp} (mg/g)	Pseudo second order				Pseudo first order		
		Q_{e2} (mg/g)	k_2 (g/(mg min))	h (mg/(g min))	R^2	Q_{e1} (mg/g)	k_1 (min^{-1})	R^2
APNs	98.3	98.6	0.043	418.0	0.9999	150.1	0.095	0.9198
PAN/APNs	97.3	98.3	0.002	18.0	0.9998	178.3	0.001	0.5026
PAN	15.7	15.7	0.012	3.0	0.9992	4.2	0.008	0.7972

Table S2. The parameters of the Langmuir model and Freundlich model

Samples	Temperature (K)	Langmuir			Freundlich	
		Q_m (mg/g)	K_L (L/mg)	R^2	$1/n$	R^2
APNs	298	698	0.323	0.9019	0.284	0.7215
	308	781	0.290	0.9229	0.294	0.7760
	318	922	0.255	0.9577	0.314	0.8182
PAN/APNs	298	556	0.022	0.9329	0.130	0.7294
	308	667	0.021	0.9218	0.158	0.7570
	318	800	0.030	0.9691	0.201	0.8368
PAN	298	333	0.003	0.9735	0.595	0.8381
